# Quality over quantity – development of communicative and social competence in dentistry at the Medical Faculty of Heidelberg

**DOI:** 10.3205/zma001456

**Published:** 2021-03-15

**Authors:** Doris Roller, Lydia Eberhard

**Affiliations:** 1 Medizinische Fakultät Heidelberg, Studiendekanat Zahnmedizin, HeiCuDent Lehrentwicklung, Heidelberg, Germany

**Keywords:** communication, dentistry, medicine, education, social behavior, attitude, feedback, interpersonal relations, intersectoral collaboration, quality of health care, professional patient relations, educational models, role playing, faculty, human, adult, male, female, germany, adult, male, female, germany

## Abstract

Given the context of implementing new licensing regulations for dentistry, this project report describes not only the current educational situation regarding communicative and social competency in dental education at the Medical Faculty of Heidelberg, but also introduces supportive and expanded measures that include medical educators and clinical staff.

Based on less-than-satisfactory skills acquisition in students and experienced practitioners, it is necesssary to develop communicative and social competence not just in university courses with few hours of instruction, but also to practice and continually improve these skills in an educational clinical setting which serves as a system for teaching and learning knowledge and skills.

## Zusammenfassung

Vor dem Hintergrund der Implementierung einer neuen Zahnärztlichen Approbationsordnung werden in diesem deskriptiven Projektbericht sowohl die aktuelle Ausbildungssituation zum Thema „Kommunikative und soziale Kompetenz“ im Fach Zahnheilkunde an der Medizinischen Fakultät in Heidelberg beschrieben als auch unterstützende und erweiterte Maßnahmen unter Einbeziehung von Dozierenden und Mitarbeitenden vorgestellt.

Anhand von ungünstiger Kompetenzentwicklungen Studierender und erfahrener Praktiker wird die Notwendigkeit aufgezeigt, kommunikative und soziale Kompetenz nicht nur in Lehrveranstaltungen mit wenigen Unterrichtsstunden zu entwickeln, sondern sie in einem lernenden System aus Lehre und Klinik im gesamten Studiums- und Arbeitsprozess zu praktizieren und kontinuierlich zu verbessern.

## Introduction

The importance of communication and social skills when providing dental care to patients is undisputed [1], [2], [3], [4]. Given that these skills can be learned [5], curricula aimed at teaching communication skills have been implemented in dental education in the German-speaking countries [6] and internationally [7], [8], [9], [10]. The National Catalogue of Competency-based Learning Objectives in Undergraduate Dental Medicine (NKLZ [http://www.nklz.de]) for Germany lists the learning objectives for students and additional skills for postgraduates. Other publications describe the importance and positive effects of training sessions for students regarding communicative competence [11], [12], [13], [14]. Most frequent are small-group sessions with standardized patients (SP) [15]. The results have been substantiated by validated measuments [16], [17], [18]. Studies have confirmed that only having contact with patients is not sufficient for students or experienced practitioners to develop communication and social skills [19]. Empathy levels were lower among experienced practitioners than among students [20]. Even with an extensive training in communication and social skills, a decline in empathy can be observed over the course of dental studies [21], [22]. Alongside the positive influences of training, there seem to be other factors at work, in the sense of a hidden curriculum, which hinder the development of robust communication and social skills [23], [24], [25].

When it comes to improving the teaching of communicative and social competence, educators play a central role, as is the case with every teaching format [26]. As a consequence, the preparation of medical educators to teach these skills and their attitudes toward them decisively shape students’ development of these skills [27].

The literature points to hindering factors [28], [29]. Studies on teamwork show the importance of cooperation [30], [31], [32]. In addition, a learning environment with a feedback culture is described as being generally encouraging of learning processes [33].

The Dental Clinic at the Heidelberg University Hospital relies on three pillars to develop communicative and social competence: teaching students, training educators, and including the clinical staff in the development of skills.

The current reason for expanding the teaching of communicative and social competence can be found in the new version of the German licensing regulations for practicing dentistry (ZApprO), set to be implemented in the 2020/21 winter semester. The new ZApprO emphasizes the importance of prevention, strengthens interdisciplinary connections, promotes practical relevance through the introduction of clinical electives (Famulatur), and encourages individual specialization by offering elective courses [34].

This paper describes the present status of the current measures being taken and introduces planned innovations for teaching and staff development.

## 1. Measures to develop communicative and social competence in students

The current program consists of two compulsory core courses in the fifth and sixth semesters. There is the option to attend a more in-depth series of seminars in the ninth semester.

The program’s preclinical courses will be expanded in response to the new ZApprO.

**Figure 1:**
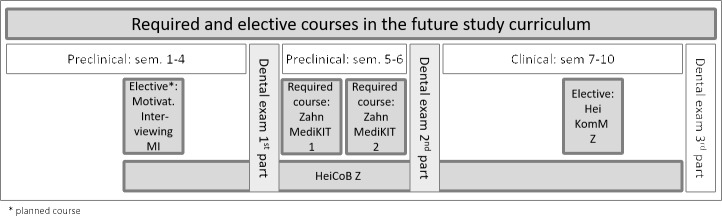
Overview of the courses on communicative and social competence in dental education at Heidelberg University.

Accompanying the practical courses, coaching support is offered. This longitudinal curriculum is presented in figure 1 (Fig. 1) and described below in more detail:

### 1.1. Semester 5: ZahnMediKIT 1

In Heidelberg, dental students first explicitly encounter the topic of communication in the fifth semester. Consultations with SP regarding a dental prosthetic are conducted according to the ZahnMediKIT teaching method (training for communication and interaction in dentistry, see below).

The l**earning objective** is to gather initial experience with patient interaction while applying knowledge acquired in the simulation courses on dental technology.

**Figure 2:**
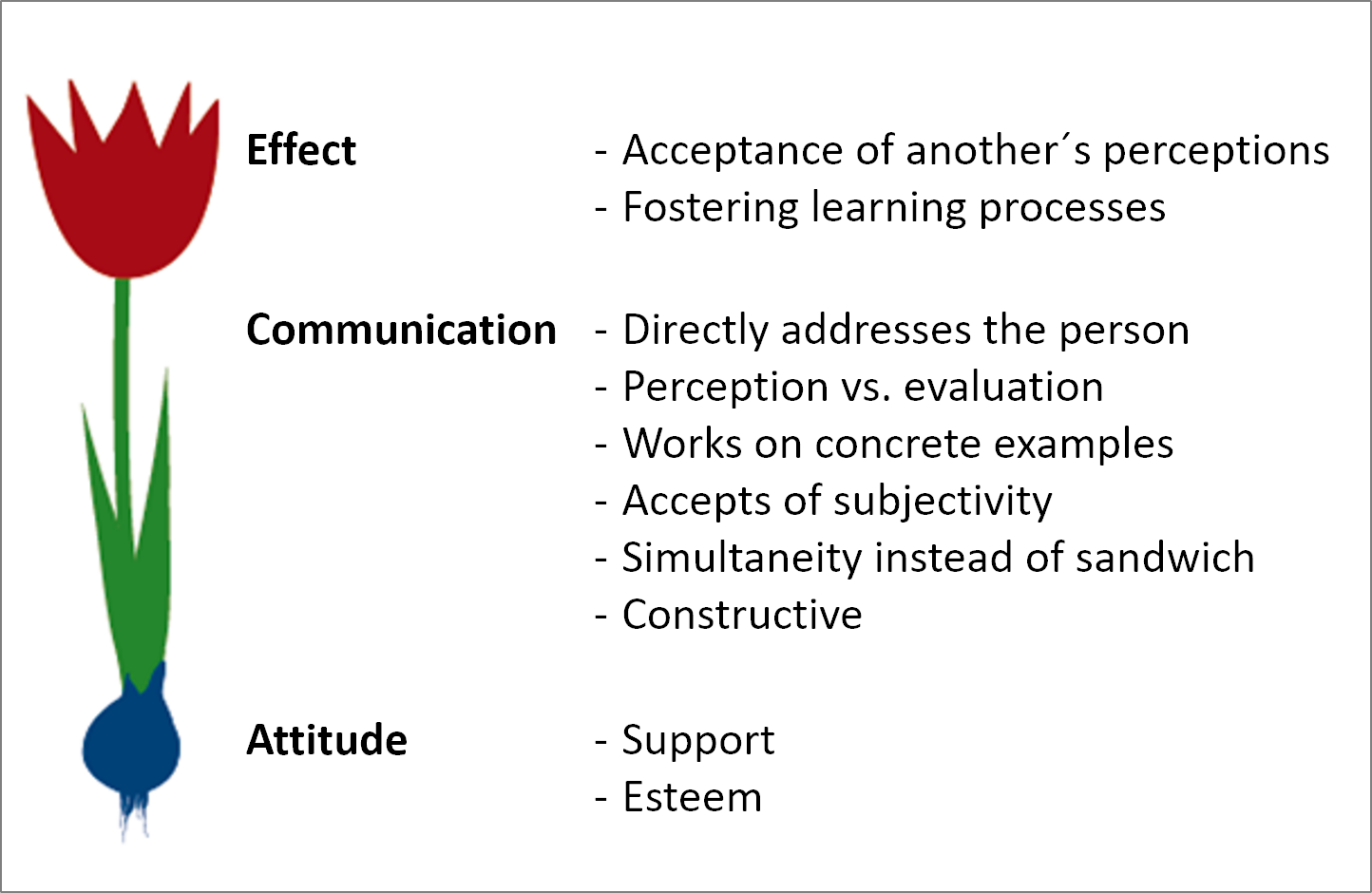
The “Heidelberg Feedback Tulip” as a model for vital feedback.

**Figure 3:**
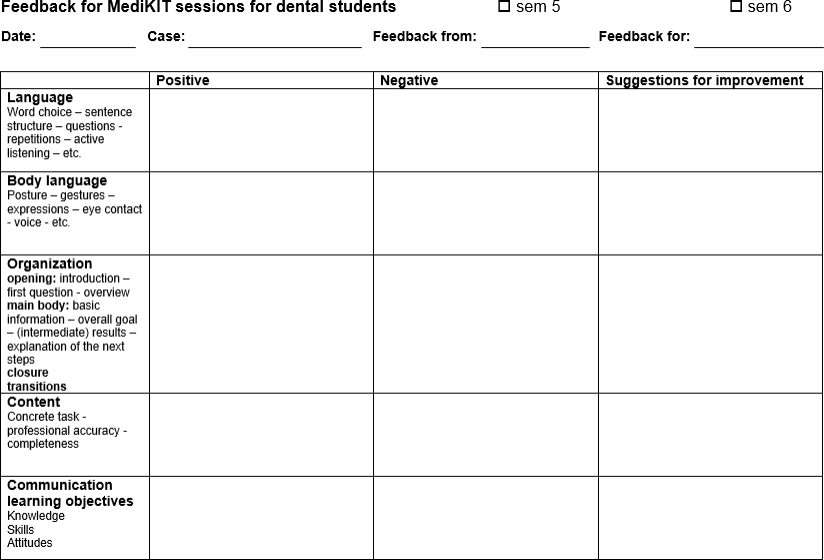
Observation worksheet for ZahnMediKIT sessions.

The ZahnMediKIT method originated from the MediKIT approach used in medical study [35] and was adapted for teaching and learning dentistry. During an introductory session, students learn about the function of feedback and how to give and receive it (see figure 2 (Fig. 2)) [36], [37], [38]. The consultations are intentionally held without any theoretical underpinnings or references to communication science. This enables the experience of the most authentic interactions possible in which a student’s personal communication style can develop and emerge on its own. The case vignettes refer to medical knowledge that has already been acquired, assign tasks (eg. preparing a patient for edentulism and the need to wear a set of complete dentures), and cover learning objectives in communication (eg, typical sensitive topics, such as recognizing and responding to fears). Students practice building relationships with patients and taking their personal concerns into consideration during the consultations. In addition, students become aware of the vast variety of possible approaches in communication by observing their fellow students. The small groups are comprised of three students. Feedback entails students watching and self-assessing video recordings of their own interactions and receiving feedback from SP, fellow students, and teachers. To support students in this process, special worksheets are used to structure the feedback highlighting communication criteria, performance of tasks, and achievement of learning objectives (see figure 3 (Fig. 3)). Alternative ways to communicate with patients are covered in the subsequent course session. Relevant parts of the conversation with the patient can be repeated in a micro-teaching setting in order to explore different communication strategies.

The courses in the fifth semester are taught by the teaching staff of the preclinical simulation course.

#### 1.2. Semester 6: ZahnMediKIT 2

In the sixth semester more complex dental cases with underlying medical conditions are handled. The **learning objectives **are developing communicative and social competence under challenging conditions, such as non-adherence, and practicing how to cooperate with other medical disciplines.

In contrast to the previous semester, all of the students have already received the preparatory information on communication and interaction. They attend a 90-minute interactive lecture that covers question-and-answer techniques, the NURSE model, the four-ears model put forth by Friedemann Schulz von Thun, and the concept of shared decision making [39], [40], [41], [42]. Case vignettes and questions on communication strategies are available in advance on the Moodle learning platform. These are meant to serve as preparation (eg, How do you recognize good/poor patient adherence?). Students decide on their own how they use the material. They are encouraged to voice their own learning objectives and to get feedback on them.

In the sixth semester, the course units are taught by specialists for internal medicine.

ZahnMediKit is a compulsory course. One session entails conducting a patient consultation and two sessions are dedicated to observing fellow students and giving peer feedback.

#### 1.3. Semesters 7-10: practice and HeiKomM Z

##### 1.3.1. Practical patient care

In the seventh through tenth semesters, students gather practical experience in treating patients in two integrated treatment courses. Students either carry out dental treatment themselves or assist their fellow students in dental procedures for 4-5 days a week. They receive informal feedback on their work by the teaching assistants in the class discussions.

##### 1.3.2. HeiKomM Z

The voluntary communication module in dentistry, referred to as HeiKomM Z (*Heidelberger Kommunikations-Modul in der Zahnmedizin*), is offered in the ninth semester.

The **learning objectives** for this module include acquiring knowledge about communication, reflecting on behaviour during interactions in the clinical setting, and practicing alternative behaviours as part of the unit on personal experience.

HeiKomM Z was developed in 2016 and has since replaced MediKIT courses in the clinical phase of study. This module encompasses seminars, a report on personal experience, and acquiring self-awareness in practical settings.

**In the first step,** four seminar sessions lasting 105 minutes each cover the following topics in interactive learning settings:

**Interaction-relevant topics** [43], [44], [45], [46]:

- personal communication styles
- structuring aids for conducting conversations
- methods to promote understanding and acceptance
- strategies for solving conflicts

**Occupation-specific topics** [47], [48], [49], [50], [51]:

- understanding the role of the dentist
- models for consulting with patients and their use in different settings
- handling emotions professionally
- managing and motivating employees

**Psychological aspects of communication** [52], [53], [54]:

- perception and construction of reality
- personality types
- intrapersonal conflict management (“Internal Team”)

**Figure 4:**
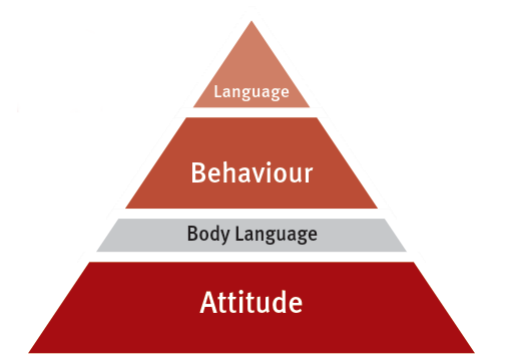
Model of communication levels.

To better anchor theoretical knowledge, important topics that have been covered in sixth-semester lectures are revisited in a learning spiral for more in-depth coverage (see figure 4 (Fig. 4)). Between seminars, students are encouraged to apply relevant knowledge in the practical dental treatment course when interacting with patients and assisting fellow students.

Individual seminars, such as “Difficult Patients” or “Conflicts in the Dental Profession,” are taught by teachers with special expertise (psychosomatic specialist; established dentist in group practice). The other topics are covered by a dentist with professional expertise in communication.

**Figure 5:**
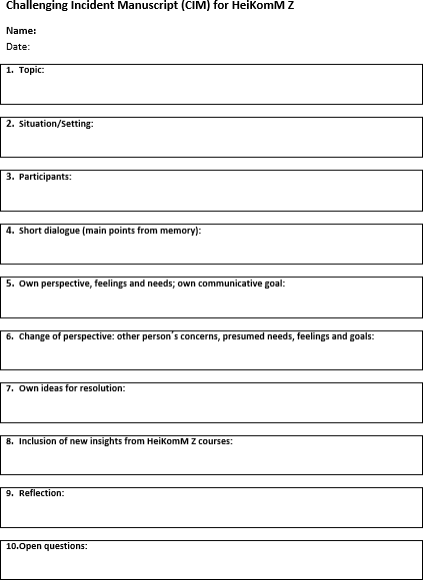
Worksheet for case processing in the personal experience unit.

**In the second step**, students document a subjectively challenging incident using a specific worksheet (Challenging Incident Manuscript, CIM, see figure 5 (Fig. 5)). This helps students analyze what happened and figure out new strategies and behaviours. They reflect on the situation and their reaction, switch perspectives, and use the lecture material to recognize better alternatives.

**In the third step**, the CIM worksheets serve as case vignettes for practical application of personal experience.

First, the case is discussed with respect to facts as well as emotions and needs in a partnered exercise. Then the cases are acted out as a role play and discussed. Material from the seminar is integrated into the activity as it unfolds.

Students may also adopt patient cases from their own clinical experience and use them anonymized in the same manner for practice.

In all of the steps of acquiring personal experience (CIM worksheet, partnered exercise, role play and discussion), the new material is connected to previously learned knowledge. This is to ensure that the students arrive at a more variegated style of interacting instead of just keeping with their familiar patterns.

When students participate in 75% of the seminars, they receive a formal certificate of attendance; active participation in the CIM and role playing is awarded another certificate. The overall challenging expectations were designed on purpose, even if this may reduce the number of students attending. As a result, it is easier to respond to students’ personal concerns, which in turn increases both the benefit to individual students and the quality of the program.

#### 1.4. Additional measures

##### 1.4.1. E-Learning

During the fifth semester, an eLearning module is offered on the subject of dental prosthetics and implants, which in addition to the technical content, also covers interaction and communication with patients who require implants.

The implicit communicative **learning objective** is to experience examples of different communicative behaviours in clinical situations.

##### 1.4.2. Module on interprofessional pediatric dentistry (MIK)

In 2019 an innovative teaching project – a module on interprofessional pediatric dentistry – was offered to students completing the ninth semester. Dental students and students of interprofessional healthcare practiced collaborating in case discussions and communication exercises. Also, an interdisciplinary part was carried out at the Dental Clinic.

The **learning objectives **entailed learning and practicing professional exchange with other disciplines and healthcare occupations and developing skills in the interaction with children and their families based on good communication.

Due to structural changes, this module has been discontinued despite receiving positive evaluations.

##### 1.4.3. Coaching in dental education (HeiCoB Z)

During their studies at the Dental Clinic, students can take advantage of a coaching program. This involves both the offer to talk and guided reflections on personal behaviours. Students develop not only the ability to self-regulate, but also experience self-efficacy. The coaching is provided by a dentist who has been trained in systemic coaching and it is not included in the evaluation of students’ academic performance.

#### 1.5. Evaluation

As of yet, MediKIT courses have been scarcely evaluated with a few questions, as they were treated as a part of overarching courses. To the question, if attending ZahnMediKIT had been worthwhile, students responded with ratings of 4.59 and 4.47 (maximum value 5) on 29 and 36 evaluations (response rate: 44.6% and 46.2%).

For HeiKomM Z the participant numbers fluctuated heavily so that an online evaluation was possible in only two of four years. The course evaluation index (LVBI) for this was 94% and 97% out of a maximum of 100%; the number of responding students was 27 and 19 (response rate: 79.4% and 43.2%).

**Figure 6:**
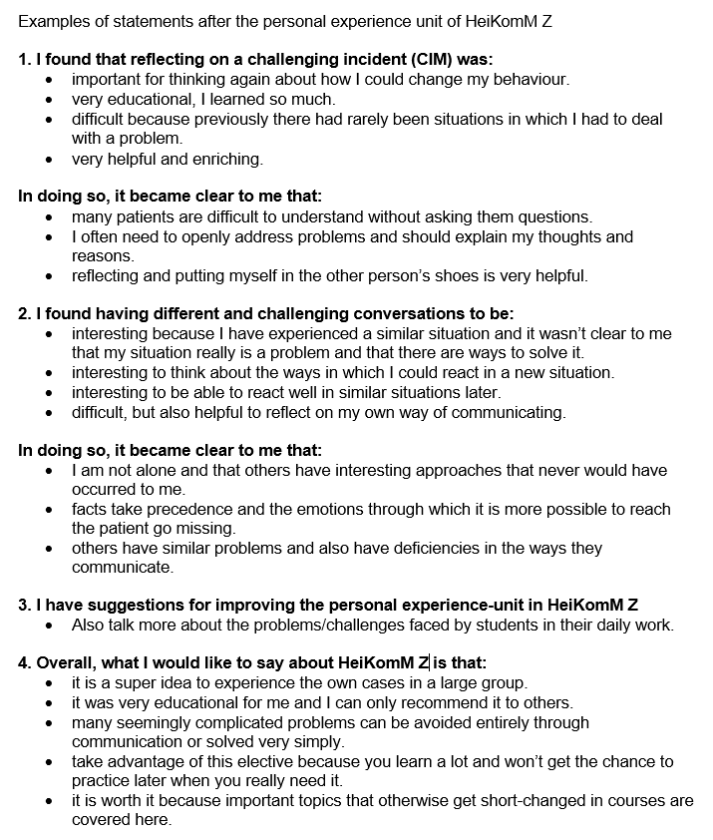
Students’ feedback after the personal experience unit in HeiKomM Z.

Statements about self-awareness were recorded separately in open-ended texts. Some examples are listed in figure 6 (Fig. 6).

#### 1.6. Skills assessment

Up to now there were no summative tests for courses on the topic of communication.

Feedback in the ZahnMediKIT sessions was understood to be formative evaluations. In the integrated clinical courses, communication skills were viewed as part of patient management and counted in the evaluation of process quality.

The evaluations for HeiKomM Z took place through feedback during exercises and a moderated discussion in the study group on personal experience.

## 2. Teaching the educators

Medical educators in dentistry and medicine go through a two-step training program of 60 instruction hours including communicative and social competence [http://www.medizinische-fakultaet-hd.uni-heidelberg.de/Schulungsaufbau.111682.0.html].

For the past three years, two additional training sessions have been required for all new dental assistants. These build on the above mentioned two-step training program, specifically focus on teaching needs in dental medicine, and were created in accordance to an intensive internal communication process. In these training sessions, topics such as the “Normality of Shortcomings” and “Errors in Judgement” are covered. Basic components include communicative and social challenges in personal interactions with students, which are practiced in typical situations.

The dentists who teach the fifth-semester ZahnMediKIT are specially trained each year. They attend a training session on feedback [36], [37], [38] and teaching strategies for imparting communication skills. They are expected to develop and internalize a professional attitude toward the effect of communication, such that in this context there is no one right answer. The learning objective is communicative behaviours that is appropriate to the context, targeted, and needs-based [40].

## 3. Communicative and social competence within the dental clinic’s system

Students learn communicative and social competence not only in curricular units on communication, but also through their initiation into the clinical setting. They observe cooperation within and between the dental departments and experience many interactions as implicit models for their own behaviours. Model learning, as described in the literature, underscores why investing in developing the models’ competence is worthwhile [55].

The related measures also promote a culture of cooperation among staff members and, as a result, fulfill the ZApprO’s demand for more networking within education.

Several innovations have already been put into place at the Dental Clinic in Heidelberg:

- For three semesters now, students in the preclinical phase of study have received structured feedback on their semester performance. The consistent inclusion of students in a transparent evaluation process led to a higher acceptance of the evaluations and – accompanied by other measures – to a better evaluation of the courses.
- Conflicts between staff members were worked out in moderated workshops. These positive experiences with communication can be drawn upon in other conflict situations and result in better resolutions in a respectful work environment.
- The permanent integration of communcation in a department’s continuing education for staff members lends weight to the topic. Patterns of communication in education and collaboration are focused on in workshops. Difficult conversations and possible reactions are discussed. In the beginning a hierarchically mixed group was reported to be a hinderance. The willingness to engage in discussions has increased after including examples from senior management.

The medium-term goal of this continuing education program is to promote exchange and make the importance of communicative and social competence visible in the form of a positive learning experience.

The courses are supplemented with individual coaching.

**Figure 7:**
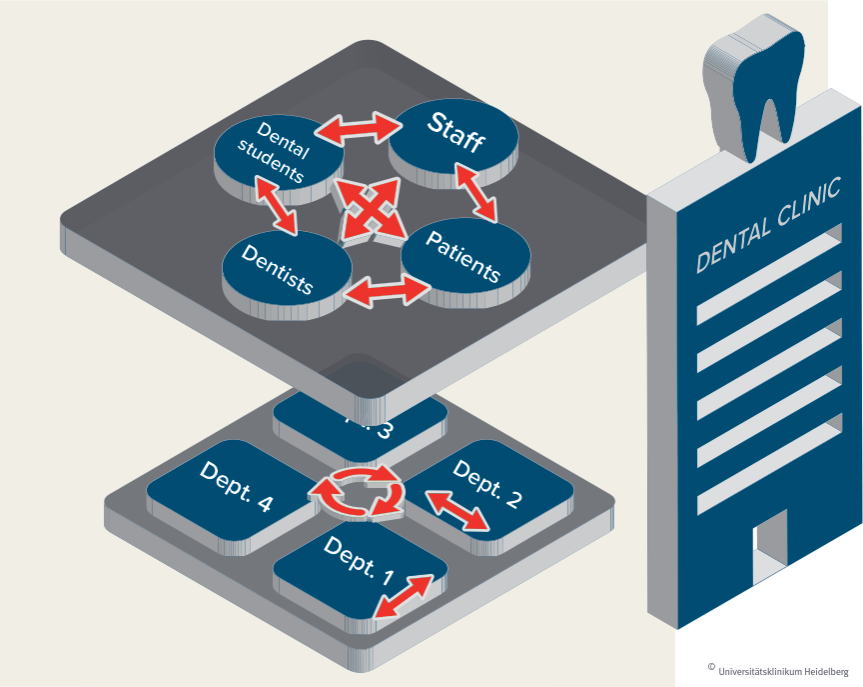
Communication contacts within the Heidelberg Dental Clinic.

The spread of the training progam to other departments creates promising conditions for sustainable problem solving and a climate of respect at the clinic, as well as in teaching and science (see figure 7 (Fig. 7)).

## 4. Development and opportunities of the new ZApprO

The sharper focus of the new ZApprO on prevention makes it easier to expand the coverage of communication as a topic in the elective courses:

In the future, the University of Heidelberg will integrate a professional consulting method into the preclinical phase and offer it as the elective course: Motivational Interviewing (MI).

MI was developed in the context of addiction prevention [56], [57] and has been sufficiently studied in the prevention of dental and periodontal disease, for instance, by means of giving instructions on good oral hygiene [58], [59]. A patient-centered and, at the same time, directive approach of this method aims to elicit the motivation of conversational partners while protecting their autonomy. With the new emphasis of the dental curriculum on prophylaxis and patient autonomy, this interview method creates favorable conditions for developing good patient adherence.

Positioning this consulting method in the preclinical phase of study is meant to raise awareness as early as possible regarding the effect communicative techniques can have when interacting with patients. In the clinical phase this knowledge can then be reviewed in subject-specific courses and applied in practice with patients. Thus, communicative behaviours are adapted for the purpose of creating a patient-dentist partnership in consultations and treatment, and these skills can be honed over the course of study.

Since elective courses will be subject to testing in the future, this will also necessitate the implementation of communicative assessment formats (eg, Objective Structured Clinical Examinations).

Evaluated testing procedures for MI have been described [60].

## 5. Discussion

The University of Heidelberg’s communication curriculum in dental education stands among other approaches that are followed elsewhere. The evaluation data which were collected are not sufficient for scientific analysis and were not intended for analysis in a study.

Curriculum development in Heidelberg relies on voluntary attendance as an alternative to compulsory courses. In the best of cases this leads to an increase in quality, but, at the same, to inevitable loss in quantity. This decision should therefore be continually revisited [61].

„Voluntariness“ is not only a central element of the elective courses defined in the new ZApprO, it is also at the core of patient-centered care [62] and forms the basis of MI, which in future will be offered as a preclinical course. We must vigilantly watch to see whether this complex interview method is well positioned so early in the curriculum. The great potential of eliciting self-awareness in the conversational partners is juxtaposed with the problem of losing out on trained resources without direct transference.

Furthermore, elective choices always imply the option not to participate. Unfavourable meeting times influence students’ decisions (HeiKomM Z is offered on Friday afternoons). Departmental training programs for staff achieve two aims: the newly acquired skills are implicitly imparted to students and, at the same time, cooperation in the team improves. A training program on topics in communication is picked up depending upon need. When no deficiencies are perceived, there has been limited interest until now.

The point is to position topics such as feedback culture, meta- and conflict communication, as well as communication of errors and cooperative culture so that their benefits can be recognized not just for individuals, but also for the entire clinical staff.

The extent to which the potential of motivational interviewing can become effective over the medium term in order to evoke participants’ needs and to achieve better communication in the clinic’s system is an interesting question for further study.

## 6. Conclusion

The new ZApprO places increased value on communicative and social competence. Various measures have already been implemented in Heidelberg’s dental curriculum to reflect this new priority.

In the future, courses will be offered even earlier in the curriculum, focus more heavily on prevention, be given more significance by being tested, and supplemented with continuing education and development opportunities for the clinical staff.

All of these measures aim to professionalize dental education and clinical practice overall through communicative and social competence in order to promote and sustain the ability to cooperate constructively using professional communicative behaviours.

## Profiles

**Institution: **Medical Faculty of Heidelberg, University Hospital

**Study program/occupational group: **Dental medicine

**Number of students per year or per semester: **Preclinical phase: 80 students per year; clinical phase: 60 students per year.

**Has a longitudinal communication curriculum been implemented? **Yes

**In which semesters is communicative and social competence taught? **Semesters 5, 6 and 9.

**Which teaching formats are used? **Small-group work, lecture, seminar, reflective individual work, role playing, eLearning.

**In which semesters is communicative and social competence assessed (formatively or necessary to pass and/or graded)? **Formatively in semesters 5, 6 and 9, but not graded or necessary to pass.

**Which assessment formats are used? **Reflection via feedback, written texts.

**Who (eg, hospital, institution) is entrusted with development and implementation? **Office of the Dean of Studies for Dentistry; Quality Management Team; Prothetics Department; Internal Medicine Department

## Current professional roles of the authors

- Doris Roller is a research assistant at the University of Heidelberg specialized in curricular development for dental education and is also active in teaching, training educators, and coaching. Her focus is on communicative and social competence.
- Lydia Eberhard is a research assistant at the Polyclinic for Dental Prosthetics at the Heidelberg University Hospital and course coordinator for the program in dental medicine. Her focus is on curricular development in traditional and digital education.

## Competing interests

The author declares that she has no competing interests.
